# CD52+ regulatory T cells and CD52-expressing sperms downregulated in couples with unexplained infertility: A case–control study

**DOI:** 10.18502/ijrm.v13i5.7159

**Published:** 2020-05-31

##  Dear Editor,

Unexplained infertility is a type of infertility with unknown reasons (1). Immunological deregulations could be considered as a reason for this disorder. The immune system is regulated by regulatory T cells (Treg) (2). Moreover, a deficiency in the function or decreasing count of Treg cells may be the reason for some unexplained infertilities. CD52 has been found to be expressed in Treg cells, sperms, and seminal plasma and has an immunosuppressive function (3). This study aimed to evaluate the expression of CD52 on Treg cells, sperm, seminal plasma, and serum in a group of couples with unexplained infertility. After the exclusion of maternal anatomic, hormonal, and paternal and maternal chromosomal abnormalities, ten unexplained infertile (case group) and five fertile couples (control group) were enrolled in this study. Semen and whole blood samples were taken from the participants. The expression of CD52 on the surface of Treg cells in peripheral blood mononuclear cells and sperm cells was measured using flow cytometry. ELISA and Western blot tests were used to examine CD52 levels in the serum and semen of the participants. Furthermore, results were analyzed using the GraphPad Prism, version 5.0 software (Inc, USA). The Mann–Whitney test was used to compare the two groups, whereas the Spearman test was used to determine their correlation. A p-value less than 0.05 was considered statistically significant. There was no difference regarding the percentage of CD52 expression in the sperm cells between the case and control groups (p = 1). Moreover, CD52 intensity in the sperm of case group men was lower than controls (p = 0.10). CD52 expression in Treg cells was lower among women (p = 0.54) and men (p = 0.07) in the case group compared to that of controls; however, it was not statistically significant. The mean fluorescent intensity of CD52 in the semen of infertile men was lower than fertile men (p = 0.10). Additionally, no differences in CD52 levels were observed in the serum (221.3 ng/ml vs. 237 ng/ml) and semen (191 ng/ml vs 175 ng/ml) among the case and control groups. Western blot results showed no differences regarding CD52 expression in the sera or semen between case and control groups (p = 0.017 and p = 0.16, respectively).

Normal pregnancy is associated with high levels of Treg cells (4, 5) whereas deficiency of Treg cells was related to unexplained infertility (6), miscarriage (7), and pre-eclampsia (8). A recent study found that Treg cells that present high levels of CD52 have also shown regulatory properties (9). This molecule is attached to its receptor named siglic-10, an inhibitory receptor that sends an inhibitory signal (10). The levels of CD52 in the serum and semen and expression of CD52 by Treg cells might play a role in unexplained infertilities. The results of this study revealed that the CD52 expression on the surface of the sperm of the infertile men was decreased compared to that of the fertile men; however, the decrease was not statistically significant. The results suggested that the decrease in CD52 expression in the sperm is a reason for the less suppressive effect of the female reproductive system during the implantation in infertile couples. Also, the percentages of CD3+ CD4+ FOXP3+ T cells expressing CD52 were decreased in infertile men than in healthy men, with a trend toward a significant difference (p = 0.07). Accordingly, these results showed that Treg cellsmay play an important role in maintaining a normal pregnancy. Moreover, the result obtained from the correlation between the expressions of CD52 in the serum and semen showed a positive significant correlation in fertile but not in infertile men. This finding suggested that the balance in CD52 expression within the serum and semen might affect the fertility outcome. The most crucial limitation of this study was the small sample size. Increasing the sample size could give a greater potential to detect significant differences. Based on the results of the present study examining the decreases levels of Treg cells expressing CD52 molecules in unexplained male infertility and the CD52 receptor (siglic-10) is suggested to understand the role of CD52 in the pathogenesis of unexplained infertility.

###  Ethical consideration

Participants in this study were assented to come in the survey according to the Ethics Committee of the school of medicine in Shiraz University of Medical sciences.

###  Acknowledgments

This study was extracted from the thesis written by one of the authors, M. Raeisi, and was supported by Shiraz University of Medical Sciences, Shiraz, Iran (Grant number: 8954 and grant number: 9386).

**Mohammad Raeisi${}^{1}$ M.Sc., Kurosh Kalantar${}^{1}$ Ph.D., Bahia Namavar Jahromi${}^{2,3}$ M.D., Behrouz Gharesi-Fard${}^{1,3}$ Ph.D.**

^1^Department of Immunology, Shiraz University of Medical Sciences. Shiraz, Iran.

^2^Department of Obstetrics and Gynecology, Shiraz University of Medical Sciences. Shiraz, Iran.

^3^Infertility Research Center, Shiraz University of Medical Sciences, Shiraz, Iran.
